# Beta_2_-Adrenoceptor Stimulation Suppresses TLR9-Dependent IFNA1 Secretion in Human Peripheral Blood Mononuclear Cells

**DOI:** 10.1371/journal.pone.0065024

**Published:** 2013-05-28

**Authors:** Tobias Hilbert, Josef Bongartz, Christina Weisheit, Pascal Knüfermann, Georg Baumgarten, Andreas Hoeft, Jens M. Poth

**Affiliations:** Department of Anesthesiology and Intensive Care Medicine, University Hospital Bonn, Germany; University of Colorado Denver, United States of America

## Abstract

**Introduction:**

IFNA1 (interferon alpha) is a key cytokine regulating the activity of numerous immune cells. Plasmacytoid dendritic cells (pDCs) as natural interferon-producing cells play critical roles as sensors of pathogens and link innate to adaptive immunity. CpG motifs within DNA sequences activating toll-like receptor 9 (TLR9) are the main stimuli eliciting IFNA1 secretion from pDCs. Adrenergic substances are capable of differentially modulating the response from various immune cells. Hence, the aim of this study was to examine how adrenoceptor stimulation influences TLR9-induced IFNA1 secretion from human pDCs.

**Methods:**

PBMCs generated from human whole blood and pDCs enriched from buffy coats were stimulated with LPS and CpG-ODN 2336 in the presence or absence of epinephrine and different adrenoceptor antagonists. Secretion of TNF and IFNA1 was measured by ELISA. Flow cytometry was used to determine efficacy of pDC enrichment and adrenoceptor expression of PBMC subsets. The influence of modified IFNA1 secretion on NK cell activity was evaluated using a colorimetric tumor cell lysis assay.

**Results:**

TLR9-induced IFNA1 secretion as well as TLR4-induced TNF secretion from PBMCs was dose-dependently attenuated by coincubation with epinephrine. Combination with different specific adrenoceptor antagonists revealed that this effect was mediated by the adrenoceptor β_2_ (ADRB2). Since flow cytometric analysis could exclude the presence of ADRB2 on pDCs, highly enriched pDCs lacked any visible impact of adrenoceptor stimulation on TLR9-induced IFNA1 release. Combination of pDCs with PBMCs restored the effect, even when they were separated by a permeable membrane. Suppression of TLR9-mediated IFNA1 secretion from PBMCs by adrenoceptor stimulation reduced the lytic activity of NK cells on K562 tumor cells.

**Conclusion:**

We provide insights into the underlying mechanisms of the interrelation between immune responses and pharmacological agents widely used in clinical practice. Our results have implications for the future treatment of human patients, in which the endogenous immune response plays a pivotal role, such as during viral infections, inflammatory diseases and cancers.

## Introduction

Dendritic cells (DCs) play a critical role as sensors of pathogens and tissue injury. They initiate and modulate adaptive immunity. Besides classical DCs (cDCs), a second set of DCs has been characterized in recent years [1]. These plasmacytoid DCs (pDCs) were shown to be the natural interferon-producing cells (IPCs), which generate the majority of circulating type I interferons (type I IFNs) upon viral infections, about 200–1000 fold more than any other blood cell [1], [2]. Opposing to cDCs, pDCs circulate in peripheral blood in which they constitute 0.5%–1.0% of human PBMCs (peripheral blood mononuclear cells) [3]. Upon activation, pDCs enter the lymph nodes to exert their functions [1], [4].

pDC-derived interferon alpha (IFNA1) is a key cytokine regulating the activity of B cells, T-helper cells (Th cells), cDCs and natural killer cells (NK cells) [1], [4]: IFNA1 induces B cell maturation into plasma cells and immunoglobulin production [5]. pDCs can induce expansion of T cell subsets and skew T cell polarization towards a Th1 phenotype in an IFNA1-dependent manner. In cDCs and monocytes, type I IFNs are required for the induction of IL12A [4], [6], and they also induce the production of IL23A and IL18, other potent Th1-driving cytokines [1]. While inducing Th1-polarization, pDC-derived IFNA1 also elicits IL10 secreting regulatory T cells (Tregs) [1], [4]. The cytolytic activity of human NK cells is strongly enhanced by IFNA1, which is paramount for antiviral and anticancer immune responses [7].

Single-stranded RNA (ssRNA) and CpG motifs within DNA sequences are the main stimuli eliciting IFNA1 secretion from pDCs. SsRNA and CpG-DNA are recognized by endolysosomal receptors. Toll-like receptor 7 (TLR7) recognizes ssRNA in pDCs [8], TLR8 is the corresponding receptor expressed in other myeloid cells, such as monocytes or neutrophils [9]. TLR9 is activated by CpG-containing DNA motifs. These can be found in bacterial, viral and, to a lesser extent, also in mammalian DNA. Synthetic CpG-containing oligodeoxynucleotides (CpG ODN) also activate TLR9 [10], [11], [12]. In pDCs, TLR7 and TLR9 signal via MyD88 and other adaptor molecules to interferon-regulatory factor 7 (IRF7), resulting in IFNA1 synthesis and secretion [13].

Due to their ability to enhance NK cell cytotoxicity and to skew T cell polarization while at the same time inducing Treg expansion and IL10 production, the role of pDCs in disease is difficult to establish. In acute viral infections, pDCs are critically required for viral control [14], such as during acute viral hepatitis or herpes simplex infections. In chronic viral diseases such as HBV and HCV infections, pDC functions seem to be suppressed [15], [16]. Recognition of self-RNA and self-DNA is the driving factor behind pDC activation in autoimmune diseases such as systemic lupus erythematosus (SLE) and psoriasis [1], [4], [17], [18]. In the latter cases, the level of pDC-derived IFNA1 correlates with the overall disease severity. In cancer, non-activated pDCs seem to suppress anti-tumor responses [19]. These pDCs remain in an inactive state either due to the lack of an appropriate stimulus or an active suppression of their activation by the tumor itself or by bystander cells.

While it is widely accepted that “stress” increases the susceptibility to viral infections and plays a role in tumor development, the underlying mechanisms are poorly understood. The endogenous stress response – mainly mediated by catecholamines like norepinephrine and epinephrine – is frequently aggravated by pharmacological interventions. One such intervention is the use of (nor-) epinephrine during cardio-circulatory resuscitation in the context of different diseases (e.g., sepsis and shock). These agents act via so-called adrenoceptors, G-protein coupled membrane receptors. These are classified as α- and β-adrenoceptors and differ in their downstream signaling mechanisms and tissue distribution. It has been shown that subsets of immune cells also express adrenoceptors and that these cells are differentially regulated by adrenergic substances [20], [21], [22], [23], [24].

In the present study, we report that TLR9-dependent IFNA1 secretion is suppressed by adrenoceptor ligands in PBMCs. We demonstrate for the first time that this effect is mediated by ADRB2 (adrenoceptor β_2_). Furthermore, we provide evidence that this ADRB2-dependent TLR9 suppression is mediated by bystander cells within PBMC and not by ADRB2 on pDCs. This observation has implications for immune reactions involving pDCs, as evidenced by decreased lysis of K562 cancer cells after ADRB2-dependent repression of TLR9-induced IFNA1 secretion.

## Materials and Methods

### Ethics Statement

All studies using primary cells were performed after written approval from the ethics committee of the medical faculty of the university of Bonn (“Ethik-Kommission der Medizinischen Fakultät”, “Rheinische Friedrich-Wilhelms Universität Bonn”) and after obtaining written informed consent from the donors. Investigations were conducted according to the principles expressed in the Declaration of Helsinki.

### Materials and reagents

RPMI 1640 was purchased from Life Technologies (CA, USA) and was supplemented with 10% heat-inactivated fetal bovine serum (FBS Superior, Biochrom AG, Berlin, Germany). Biocoll Separating Solution (1.077g/ml) was also acquired from Biochrom AG. 24- and 96-well flat-bottom plates were obtained from Greiner Bio-One (Frickenhausen, Germany), 24- Transwell Permeable Support Plates (3413, 0.4 µm pore size, polycarbonate membrane, 6.5 mm insert diameter) were purchased from Corning Incorporated Life Sciences (MA, USA). Epinephrine-HCl (E4642), Propranolol-HCl (P0884), Metoprolol-Tartrate (M5391), ICI118,551-HCl (I127), Urapidil-HCl (U100) and RX821002-HCl (R9525) were provided by Sigma-Aldrich (MO, USA) and dissolved in sterile water. CpG ODN 2336 (tlrl-2336) was purchased from InvivoGen (CA, USA), lipopolysaccharide (LPS) was from Sigma-Aldrich (L4130).

Reagents for Magnetic Activated Cell Sorting (MACS) and Fluorescent Activated Cell Sorting (FACS) (CD304 Microbead Kit, mouse anti-human-CD14-PerCP antibody, mouse anti-human-CD303-PE antibody, mouse anti-human-CD304-APC antibody and FcR blocking reagent) were obtained from Miltenyi Biotec (Bergisch Gladbach, Germany) and BD Biosciences (CA, USA) (mouse anti-human-CD123-Brilliant-Violet421 antibody), respectively. Additional antibodies were purchased from Abcam (Cambridge, USA): Anti-ADRB2 antibody (polyclonal rabbit, Abcam ab36956), anti-rabbit-IgG antibody (goat, Abcam ab6717) and rabbit IgG-ChIP Grade (isotype control, Abcam ab37415). FACS analysis was performed on a BD FACSCanto II flow cytometer. FACS data was acquired using BD FACSDiva (version 6.1.2) and analyzed using FlowJo (version 10.0.5) software. Assay buffer for FACS and MACS was prepared by adding 0.5% FBS and 2 mM EDTA (0.5 M, pH 8.0, Merck KGaA, Germany) to PBS (pH 7.4, Life Technologies).

### Cell culture

Whole blood samples (15 IU/ml heparin added) were drawn from healthy donors and peripheral blood mononuclear cells (PBMCs) were isolated by Ficoll density gradient centrifugation. Cells were seeded in cell culture medium (RPMI 1640 containing 10% FBS) into 96-well plates and stimulated with either LPS or CpG ODN 2336 in the presence or absence of epinephrine and different adrenoceptor antagonists for 24 hours (as indicated). PBS was used as vehicle control. The cell culture supernatants were collected and stored at −20°C for later analysis.

To enrich plasmacytoid dendritic cells (pDCs), PBMCs were isolated by Ficoll density gradient centrifugation from freshly prepared buffy coats from healthy human donors provided by the Institute for Experimental Hematology and Transfusion Medicine of the University Hospital Bonn (Germany). PDCs were separated by magnetic labeling of CD 304^+^ cells using MACS technique according to the manufacturer's instructions. The efficacy of enrichment was determined by staining cells for CD303 and counting CD303^+^ cells via flow cytometry.

pDCs were stimulated in RPMI 1640 containing 10% FBS with PBS as vehicle control or CpG ODN 2336 in the presence or absence of epinephrine for 24 hours. In some experiments, PBMCs were added to the enriched pDCs at a ratio of 1∶10 (pDCs∶PBMCs). For transwell experiments, pDCs were seeded into the upper compartment of a two-chamber transwell system and PBMCs were added to the lower chamber, followed by incubation with PBS or CpG ODN 2336 with or without epinephrine. After 24 hours, the cell culture supernatants were collected and stored at −20°C for later analysis.

Commercially available K562 cells (ATCC line CCL-243) were a kind gift from the Institute of Clinical Chemistry and Pharmacology of the University Hospital Bonn. They were cultured in RPMI 1640 containing 10% FBS.

Cell culture was carried out in a standard cell culture incubator (37°C in a 5% CO2 humidified atmosphere).

### Measurement of cytokines in cell culture supernatant

TNF and IFNA1 levels in the cell culture supernatant were quantified using commercially available ELISA kits (BD Biosciences (CA, USA) and eBioscience (CA, USA), respectively) according to manufacturer instructions.

### Cell viability measurement

To asses cytotoxic effects of adrenoceptor agonist and antagonist treatment, we used the “CellTiter-Blue Cell Viability Assay” (Promega, Madison, WI, USA). After stimulating cells as indicated, resazurin solution was added. 3 hours later, the turnover of resazurin to resorufin was measured using a fluorescent plate reader (EnVision, PerkinElmer, Waltham, MA, USA).

### Detection of ADRB2 via flow cytometry

PBMCs were stained for CD123, CD14 and CD304 according to the manufacturer's instructions and additionally incubated with either anti-human-ADRB2 antibody (3 µg/10^−6^ cells) or isotype control (3 µg/10^−6^ cells), supplemented with FcR Blocking Reagent (1∶7), for 10 minutes at 4°C. After washing with FACS buffer, cells were stained with a FITC-labeled secondary anti-rabbit-IgG antibody (1∶300) for 10 minutes (4°C), followed by another wash step and by readout on flow cytometer.

### NK cell activity assay (tumor cell lysis assay)

The use of the leukemic cancer cell line K562 for NK cell mediated lysis has been described before [25], [26].

Here, preconditioned cell culture supernatants were generated by incubating PBMCs with PBS or CpG ODN 2336 in the presence or absence of epinephrine for 24 hours. Harvested supernatants were stored at −80°C for later use.

After measurement of IFNA1, supernatants were individually diluted depending on the maximum IFNA1 level achieved by incubation with CpG ODN to avoid supramaximal stimulation of NK cells. Of note, supernatants derived from the same donor (after incubation with PBS, CpG ODN and CpG ODN plus epinephrine) were diluted using the same dilution factor. Freshly prepared PBMCs were pretreated with these diluted supernatants for 4 hours. To avoid any direct effects of contained epinephrine on NK cell activity, propranolol was added (10^−7^ M) to the supernatants. In a following step, K562 cells were added. Previous titration experiments determined the optimal ratio of K562 cells to PBMCs at 1∶12.5. During coincubation for 2 hours, the NK cells contained in the PBMCs lysed the K562 cells. Finally, the level of lactate dehydrogenase in the cell culture supernatant was measured using the LDH Cytotoxicity Kit II (PromoCell, Germany) according to manufacturer instructions. The spontaneous lysis of PBMCs and K562 cells was subtracted.

Figures show mean lytic activity (expressed as percentage of total cell lysis achieved by adding cell lysis buffer) from one representative experiment out of three independent experiments using preconditioned supernatants and NK cells from PBMCs from at least 6 different donors.

### Statistics

Data are presented as the mean of triplicates ± SEM of one representative experiment out of at least three independent experiments with PBMCs derived from different donors and pDCs enriched from different buffy coats, respectively. Statistical analysis was performed by two-sided unpaired Student's t-test, and data was visualized using GraphPad PRISM (La Jolla, CA, USA). The alpha level was set to 5%.

## Results

### ADRB2 stimulation of human PBMCs suppresses secretion of TNF upon TLR4 stimulation

**Figure 1:**
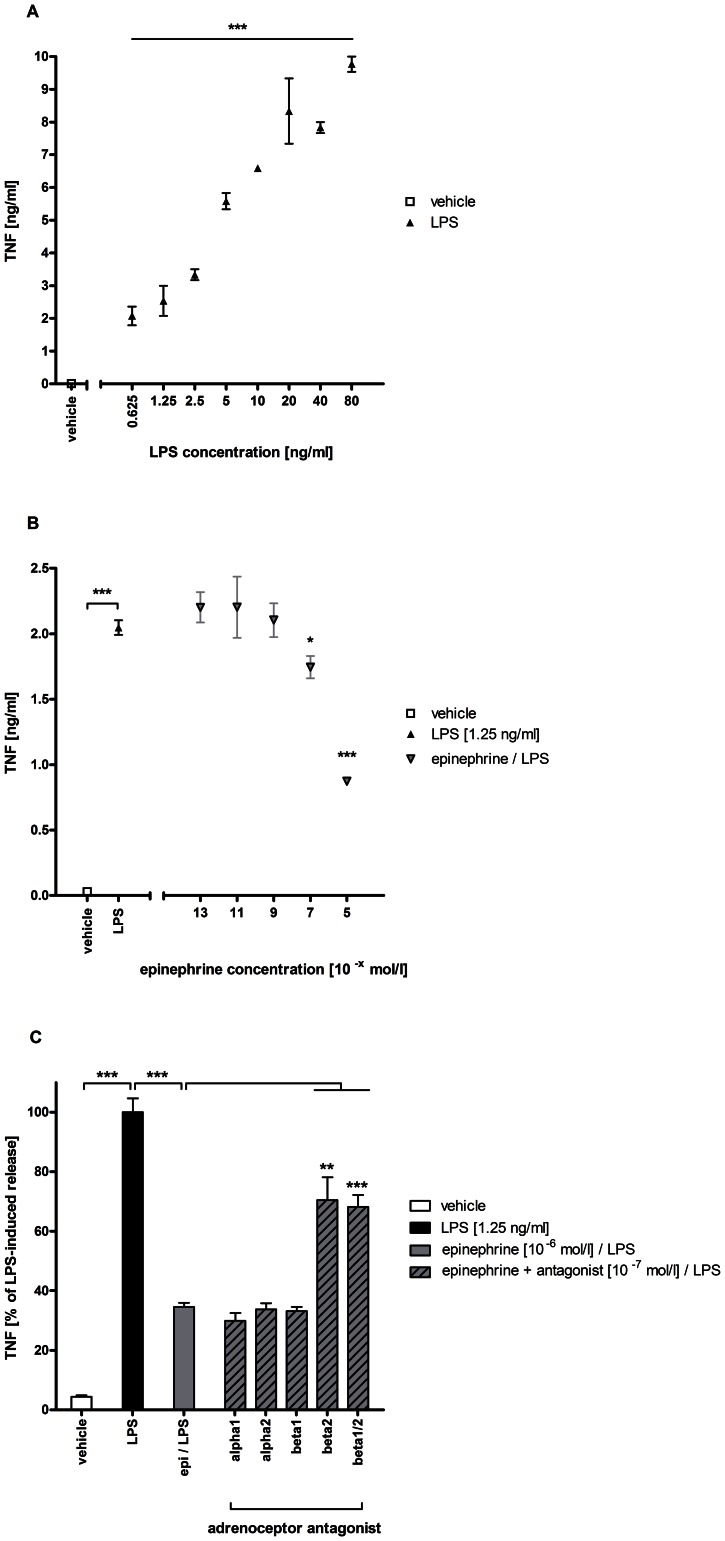
Effect of ADRB2 stimulation on TLR4-mediated TNF release in human PBMCs. PBMCs were generated from freshly-drawn blood from healthy human donors. (A) After stimulation with PBS (vehicle) or LPS in increasing concentrations (0.625–80 ng/ml) for 24 hours, TNF release into the supernatant was measured by ELISA; p<0.005 for LPS (each concentration) vs. vehicle. (B) PBMCs were stimulated with PBS (vehicle), LPS (1.25 ng/ml) or LPS in the presence of epinephrine in increasing concentrations (10^−13^–10^−5^ mol/l). After 24 hours, TNF release into the supernatant was measured by ELISA; p<0.005 for LPS vs. vehicle, p<0.05 for LPS vs. epinephrine (10^−5^) plus LPS and p<0.01 for LPS vs epinephrine (10^−7^ mol/l) plus LPS. (C) PBMCs were stimulated with PBS (vehicle), LPS (1.25 ng/ml) or LPS in the presence of epinephrine (10^−6^ mol/l) and different adrenoceptor antagonists (10^−7^ mol/l). After 24 hours, TNF release into the supernatant was measured by ELISA. Data is presented as percentage of LPS-induced TNF secretion. Statistical comparisons are indicated by brackets. epi = epinephrine; alpha1 = α_1_-adrenoceptor antagonist (urapidil); alpha2 = α_2_-adrenoceptor antagonist (RX821002); beta1 = β_1_-adrenoceptor antagonist (metoprolol); beta2 = β_2_-adrenoceptor antagonist (ICI118,551); beta1/2 = β_1/2_-adrenoceptor antagonist (propranolol). * p<0.05; ** p<0.01; *** p<0.005.

Human PBMCs were stimulated with increasing concentrations of LPS (0.5–100 ng/ml). After 24 hours, TNF levels in the cell supernatant were measured by ELISA. The release of TNF was concentration-dependent (Fig. 1A), showing a significant increase in TNF secretion with 0.625 ng/ml of LPS. 1.25 ng/ml of LPS were used for subsequent experiments.

When PBMCs were co-incubated with LPS (1.25 ng/ml) and various concentrations of epinephrine, TNF release was attenuated (Fig. 1B). This effect was dose-dependent. Epinephrine concentrations below 10^−9^ M had no effect on LPS-induced TNF release. There was no TNF release detectable after incubation with epinephrine or adrenoceptor antagonists alone.

In further experiments, highly selective α- and β-adrenoceptor antagonists were combined with epinephrine to investigate which adrenoceptor mediated the observed effect. Neither the β_1_-antagonist metoprolol nor the α-antagonists urapidil (α_1_-selective) nor RX821002 (α_2_-selective) reduced the epinephrine-mediated suppression of TNF release, while the non-specific β-blocking agent propranolol and the β_2_-specific agent ICI118,551 did, identifying the β_2_-adrenoceptor (ADRB2) as mediator of epinephrine-dependent suppression of TNF release (Fig. 1C). Remarkably, suppression of TLR4-induced TNF release could be observed after short incubation times (i.e., 4 hours). Dose response experiments showed that blocking capacity was most effective for concentrations 10-fold lower than that of epinephrine (data not shown).

### ADRB2 stimulation attenuates TLR9-mediated IFNA1 secretion in human PBMCs

**Figure 2:**
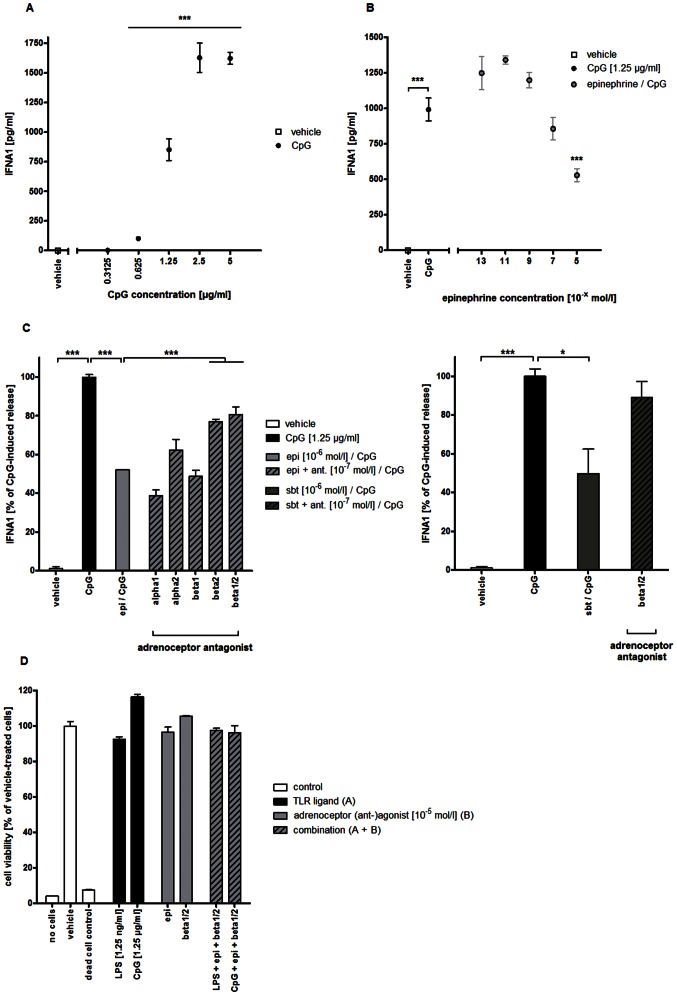
Effect of ADRB2 stimulation on TLR9-mediated IFNA1 release in human PBMCs. PBMCs were generated from freshly-drawn blood from healthy human donors. (A) After stimulation with PBS (vehicle) or CpG ODN 2336 in increasing concentrations (0.3125–5 µg/ml) for 24 hours, IFNA1 release into the supernatant was measured by ELISA; p<0.005 for CpG (0.625–5 µg/ml) vs. vehicle. (B) PBMCs were stimulated with PBS (vehicle), CpG ODN 2336 (1.25 µg/ml) or CpG ODN in the presence of epinephrine in increasing concentrations (10^−13^–10^−5^ mol/l). After 24 hours, IFNA1 release into the supernatant was measured by ELISA; p<0.005 for CpG vs. vehicle and vs. epinephrine (10^−5^ mol/l) plus CpG. (C) PBMCs were stimulated with PBS (vehicle), CpG ODN 2336 (1.25 µg/ml) or CpG ODN in the presence of epinephrine (left panel) or salbutamol (10^−6^ mol/l, right panel) and different adrenoceptor antagonists (10^−7^ mol/l). After 24 hours, IFNA1 release into the supernatant was measured by ELISA. Data is presented as percentage of CpG ODN-induced IFNA1 secretion; p<0.005 for CpG vs. vehicle and vs. epinephrine plus CpG; p<0.05 for CpG vs. salbutamol plus CpG. Otherwise, statistical comparisons are indicated by brackets. (D) PBMCs were stimulated with PBS (vehicle), LPS (1.25 ng/ml), CpG ODN 2336 (1.25 µg/ml), epinephrine (10^−5^ mol/l) or propranolol (10^−5^ mol/l) or with a combination. For dead cell control, cell death was induced by adding DMSO to the cell culture medium (30 vol%). For assessment of spontaneous conversion of resazurin to resorufin, a control without cells (‘no cells’) was included. After 24 hours, resazurin solution was added and cell viability was determined after additional 3 hours by quantifying the conversion of resazurin to resorufin using a fluorescent plate reader. Fluorescence signal from vehicle-treated cells was set as ‘100% viability’. CpG = CpG ODN 2336; epi = epinephrine; sbt = salbutamol; alpha1 = α_1_-adrenoceptor antagonist (urapidil); alpha2 = α_2_-adrenoceptor antagonist (RX821002); beta1 = β_1_-adrenoceptor antagonist (metoprolol); beta2 = β_2_-adrenoceptor antagonist (ICI118,551); beta1/2 = β_1/2_-adrenoceptor antagonist (propranolol). * p<0.05; *** p<0.005.

After incubation with increasing concentrations (0.3125–5 µg/ml) of CpG ODN 2336 for 24 hours, human PBMC released IFNA1 in a dose-dependent manner. Significantly elevated levels of IFNA1 were detected after stimulation with 0.625 µg/ml CpG ODN. Maximal IFNA1 secretion was observed with CpG concentrations of 2.5 µg/ml or higher (Fig. 2A). 1.25 µg/ml of CpG ODN was used for subsequent experiments.

To examine whether this TLR9-mediated IFNA1 secretion is also influenced by adrenoceptor stimulation (similar to TNF secretion after TLR4 stimulation), we combined CpG ligation with epinephrine in increasing concentrations. Significant reduction of CpG-induced cytokine secretion into the supernatant was observed with epinephrine concentrations of 10^−6^ M or higher (Fig. 2B and 2C). Similar to TLR4 stimulation, the combination of epinephrine with the β_2_-blocking agents propranolol and ICI118,551 in 10-fold lower concentrations than epinephrine led to significant attenuation of the epinephrine-mediated suppression of IFNA1 secretion (Fig. 2C, left panel). Blockade of adrenoceptors other than the ADRB2 had no such effect. Neither epinephrine nor adrenoceptor antagonists alone exerted any detectable effect on IFNA1 secretion. The combination of CpG ligation with the selective β_2_-adrenoceptor agonist salbutamol likewise resulted in a significant attenuation of IFNA1 release (Fig. 2C, right panel).

Longterm treatment (24 hours) of human PBMCs with either TLR ligands, with adrenoceptor agonist/antagonist or with the combination of both did not have any negative influence on the viability of cells (even when used in high concentrations) as determined by CellTiterBlue™ assay (Fig. 2D).

### ADRB2 is not expressed by human pDCs

**Figure 3:**
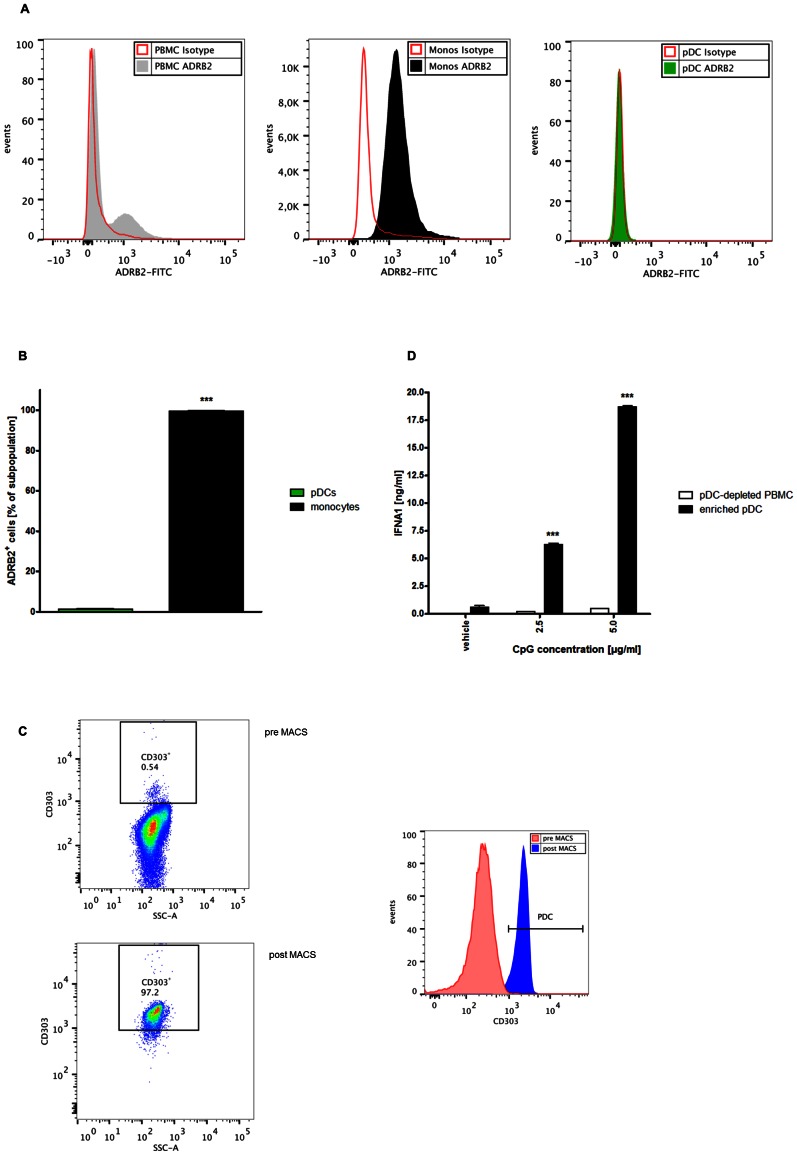
Expression of ADRB2 in human PBMCs, enrichement of pDCs. (A, B) PBMCs were generated from freshly-drawn blood from healthy human donors. After staining with antibodies against CD123, CD304, CD14 and ADRB2 or an isotype control, cells were incubated with FITC-labeled secondary anti-rabbit-IgG antibody, and ADRB2 expression was assessed by flow cytometry. (A) Histograms showing the fluorescence signal for ADRB2-FITC in PBMCs and PBMC subsets. B) Percentage of ADRB2^+^ cells within pDC- or monocyte-subpopulations within PBMC. (**C**, **D**) PBMCs were isolated from freshly prepared buffy coats from healthy human donors and pDCs were subsequently enriched by MACS technique. (**C**) Cells were labeled with anti-human-CD303 antibody, and the frequency of CD303^+^ cells was determined by flow cytometry. Dot plots show percentage of CD303^+^ cells before (upper panel) and after MACS procedure (lower panel) (gated on live cells). Right: Histogram showing the fluorescence signal for CD303 before and after MACS procedure. (**D**) Following MACS procedure, enriched pDCs and the pDC-depleted PBMCs were stimulated with PBS (vehicle) or CpG ODN 2336 (2.5 or 5.0 µg/ml). After 24 hours, IFNA1 release into the supernatant was measured by ELISA; p<0.005 for enriched pDCs vs. pDC-depleted PBMCs. CpG = CpG ODN 2336; SSC = side scatter. *** p<0.005.

We wondered whether the suppressive effect of ADRB2 stimulation was mediated by a direct influence on the pDC as the IFNA1 producing cell itself. To gain insight, we performed flow cytometric analysis of the ADRB2 expression within human PBMC subsets (Fig. 3A). As shown by surface staining, we could exclude the presence of the ADRB2 on pDCs, while other cell fractions within PBMCs (i.e., monocytes) showed a distinct signal for this receptor (Fig. 3B).

We generated an enriched pDC fraction from PBMCs by positive selection of CD304^+^ cells using magnetic activated cell sorting (MACS). Flow cytometric analysis showed that this procedure consistently enriched pDC fractions to more than 90% (compared to 0.5%–1.0% in crude PBMC) (Fig. 3C). As expected, stimulation of these enriched pDCs with CpG ODN 2336 for 24 hours resulted in dose dependent IFNA1 release into the cell supernatant (Fig. 3D). Due to the higher prevalence of pDCs, IFNA1 secretion was much more pronounced compared to CpG stimulation of crude PBMCs. PBMCs depleted from pDCs in contrast lacked any appreciable IFNA1 release, indicating that pDCs are the main source of IFNA1 secreted upon TLR9 stimulation.

### Bystander cells mediate suppression of IFNA1 secretion upon adrenoceptor stimulation

**Figure 4:**
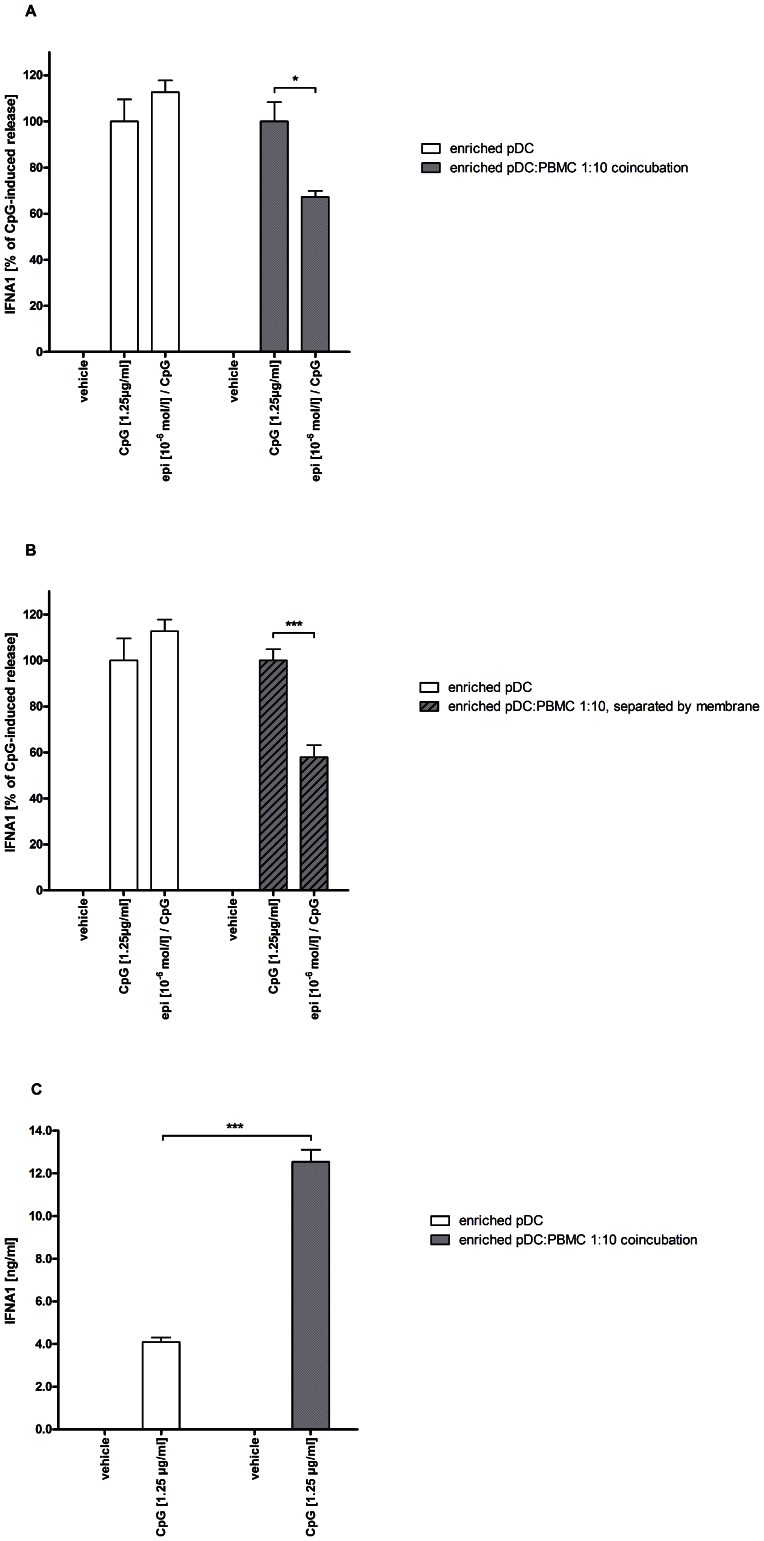
Interaction of enriched pDCs and PBMCs. PBMCs were generated from freshly-drawn blood from healthy human donors. PDCs were enriched by MACS technique from PBMCs derived from freshly prepared buffy coats from healthy human donors. (A) Enriched pDCs (white bars) or pDCs supplemented with PBMCs (pDC∶PBMC ratio 1∶10, grey bars) were stimulated with PBS (vehicle), CpG ODN 2336 (1.25 µg/ml) or CpG ODN in the presence of epinephrine (10^−6^ mol/l). After 24 hours, IFNA1 release into the supernatant was measured by ELISA. Data is presented as percentage of CpG ODN-induced IFNA1 secretion. (B) In the same experiment, enriched pDCs (white bars) were compared to enriched pDCs seeded into the upper compartment of a two-chamber transwell system containing PBMCs in the lower compartment (pDC∶PBMC ratio 1∶10, hatched bars). Cells were stimulated with PBS (vehicle), CpG ODN 2336 (1.25 µg/ml) or CpG ODN in the presence of epinephrine (10^−6^ mol/l). After 24 hours, IFNA1 release into the supernatant was measured by ELISA. Data is presented as percentage of CpG ODN-induced IFNA1 secretion. (C) Absolute IFNA1 levels of corresponding conditions in (A). CpG = CpG ODN 2336; epi = epinephrine. * p<0.05; *** p<0.005; statistical comparisons are indicated by brackets.

Since we could not detect ADRB2 on pDCs (see above, fig. 3), we were expecting the release of IFNA1 not to be modulated by epinephrine in highly purified pDCs. Indeed, co-incubation of CpG-stimulated pDCs with epinephrine had no suppressive effect on IFNA1 secretion. The suppression of IFNA1 could only be observed after adding PBMCs to the enriched pDC fraction (pDC∶PBMC ratio 1∶10) (Fig. 4A). This effect was not mediated by direct cell-cell contact, since it could still be observed in pDCs separated from bulk PBMCs by a permeable membrane (0.4 µm pore size) (Fig. 4B). This suggests that the attenuation of IFNA1 secretion is mediated by a humoral factor released from PBMCs upon ADRB2 stimulation. In the absence of epinephrine, the presence of PBMCs raised the overall IFNA1 level upon TLR9 ligation more than three-fold compared to stimulation of ‘pure’ enriched pDCs (Fig. 4C) (see also discussion).

Incubation with epinephrine for 24 hours did not down-regulate the expression of TLR9 compared to control conditions, as assessed by quantitative real-time PCR (data not shown).

### ADRB2 stimulation attenuates NK cell mediated tumor cell lysis by repression of IFNA1 release

**Figure 5:**
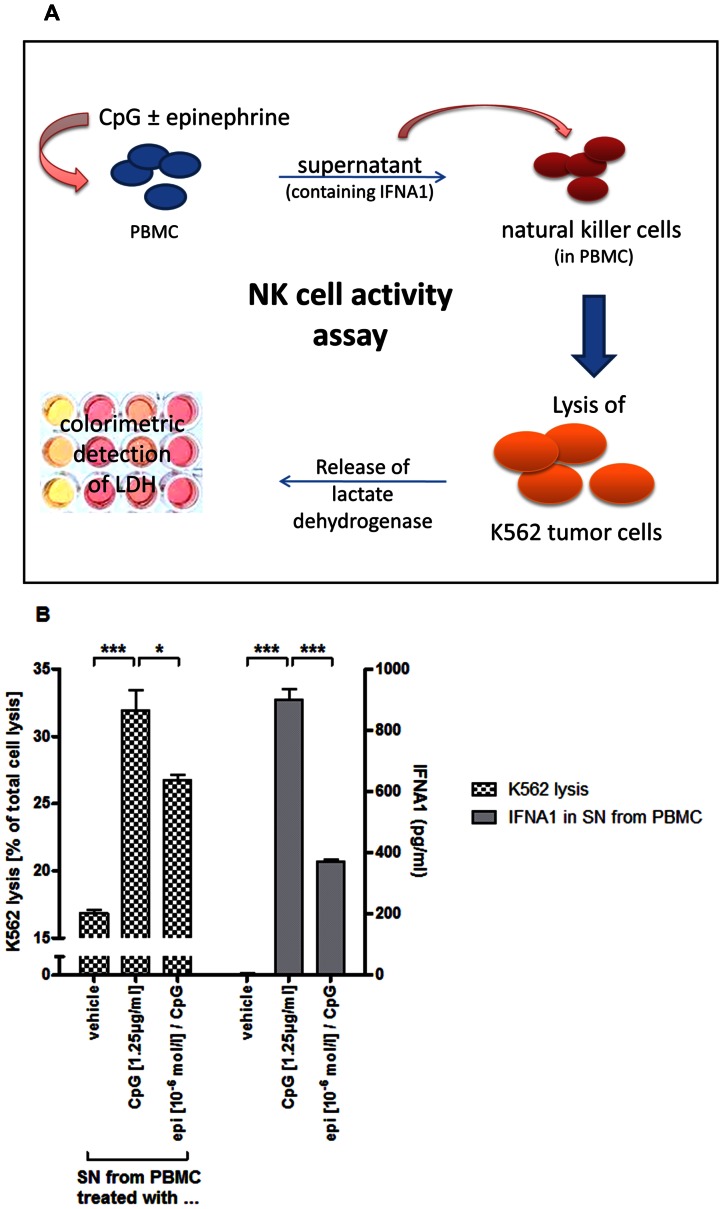
Effect of ADRB2-mediated suppression of IFNA1 release on NK cell activity. (A, B) Preconditioned cell culture supernatants were generated by incubating PBMCs with either PBS or CpG ODN 2336 in the presence or absence of epinephrine for 24 hours. Individually diluted supernatants (see Materials and Methods) were used to pretreat freshly prepared PBMCs. Propranolol was added to diminish direct effects of epinephrine on NK cells. After 4 hours, K562 cells were added (K562∶PBMC ratio 1∶12.5) and the amount of lactate dehydrogenase in the cell culture supernatant was determined after additional 2 hours by a colorimetric assay. (B) Lysis of K562 cells by PBMCs pretreated with supernatants (SN) from PBMCs incubated with either PBS (vehicle), CpG ODN 2336 (1.25 µg/ml) or CpG ODN in the presence of epinephrine (10^−6^ mol/l) (dotted bars). Data is presented as percentage of total cell lysis. The amount of IFNA1 in the preconditioned supernatants from PBMCs was measured by ELISA (solid bars). Statistical comparisons are indicated by brackets. CpG = CpG ODN 2336; epi = epinephrine. * p<0.05; *** p<0.005.

The lytic activity of NK cells is greatly enhanced in the presence of IFNA1. We examined the *in vitro* lysis of K562 cells (immortalized myelogenous tumor cell line) by NK cells after priming with conditioned cell supernatant from PBMCs being previously stimulated with CpG ODN 2336 in the presence or absence of epinephrine. The lytic activity of NK cells was measured by detecting LDH in the supernatant, which was released from K562 cells upon lysis (Fig. 5A).

When NK cells were primed with IFNA1-containing supernatant from PBMCs being stimulated with CpG alone, their lytic activity was almost doubled compared to the use of supernatant from PBS-stimulated PBMCs (Fig. 5B). Suppression of TLR9-mediated IFNA1 secretion by simultaneous adrenoceptor stimulation reduced this enhancement significantly. This correlates with the reduction of IFNA1 secretion from PBMCs by epinephrine.

## Discussion

pDCs selectively express TLR7 and TLR9 and within human immune cells, IFNA1 formation is limited to pDCs. Therefore, within human PBMCs, TLR9 ligand-induced (i.e., CpG ODN-induced) IFNA1 secretion is mediated by stimulation of TLR9 on pDCs. For the first time, we provide detailed insight into epinephrine-mediated modulation of TLR9 signaling on these cells showing that epinephrine inhibits TLR9-induced IFNA1 release from PBMCs. We also show that epinephrine suppresses TLR4-induced TNF release from primary human PBMCs. Pharmacologic studies utilizing specific adrenoceptor agonists and antagonists revealed that both effects – suppression of TLR4-mediated TNF release and suppression of TLR9-mediated IFNA1 release – were mediated by ADRB2. ADRB2-mediated IFNA1 suppression was lost in highly purified pDCs. Using flowcytometric single cell analysis, ADRB2 expression was confirmed for monocytes, but not for pDCs within PBMCs. In agreement with this observation, ADRB2-mediated modulation of TLR9 signaling in pDCs required the presence of other PBMC subsets as evidenced by add-back experiments. Modulation of TLR9-dependent pDC activation by PBMCs did not require cell-cell contact, as demonstrated by transwell experiments. Lastly, we provide evidence for possible down-stream effects of adrenoceptor-mediated suppression of pDC function showing suppression of IFNA1-dependent increased tumor cell lysis by epinephrine.

Our study adds to previous evidence from others [20] that adrenoceptor signaling suppresses TLR4 signaling. The results presented here confirm recent studies by Kizaki et al. [27], [28] and by Wang et al. [29] that this effect is mediated by ADRB2.

Other than the effects on TLR4 signaling, the interplay of adrenoceptors with TLRs involved in antiviral and antitumor immune responses has not been studied extensively. Collado-Hidalgo et al. published that norepinephrine suppresses type I interferon expression via PKA (protein kinase A) [30]. While their study added substantial data about catecholamine-mediated immuno-suppression, it lacked the identification of the adrenoceptor subtype involved in suppression of TLR9 signaling. We were able to identify ADRB2 as being the relevant adrenoceptor. However, we provide convincing evidence that ADRB2 is not expressed on pDCs. In agreement with this finding, we could only observe catecholamine-dependent pDC suppression when PBMCs other than pDCs were present. We would like to point out that Collado-Hidalgo enriched pDCs to about 40% and therefore always co-cultured them in the presence of contaminating PBMCs. While we believe their conclusion that norepinephrine exerts a direct effect on pDCs has to be questioned, their original data is actually in agreement with ours. Based on our own data, we propose two possible mechanisms for catecholamine-mediated pDC suppression within PBMCs: First, upon ADRB2 stimulation, PBMC subsets might release a humoral factor directly suppressing pDCs. This was shown to be the case for suppression of TLR9 signaling in pDCs by monocytes in the presence of bacterial cell wall products [3]. Furthermore, a whole set of surface receptors inhibiting IFNA1 release (e.g., Siglec-H, NKp44, BDCA2 and more) was recently identified on pDCs [1]. It is not known, whether these surface molecules have natural ligands they interact with. Second, IFNA1 release from pDCs is enhanced by PBMCs, presumably by cytokine signaling. This is in accordance with findings of other authors [31]. ADRB2 signaling could suppress these paracrine effects.

Liu and colleagues demonstrated that activated pDCs are potential inducers of antitumor responses mediated by NK cells and T cells [7]. We used an *in vitro* assay to investigate possible effects of epinephrine on the modulation of NK cell- dependent tumor cell lysis by pDCs. While the addition of CpG ODN-treated cell culture supernatants enhanced tumor cell lysis, this effect was attenuated when the supernatant was taken from cells being co-incubated with epinephrine and TLR9 ligands. Since direct effects of adrenoceptor stimulation on NK cell activity have been described [32], we added propranolol with cell culture supernatants in all cases to diminish direct effects of epinephrine on NK cells.

Another key function of pDCs is the initiation of appropriate antiviral immune responses [1], [4]. Collado-Hidalgo demonstrated that norepinephrine facilitates replication of HIV by suppression of type I interferon responses [30]. It is conceivable that similar effects will be observed in future studies of type I interferons, pDCs and their interplay with catecholamines.

It is widely accepted knowledge that the neuroendocrine system modulates immune responses via its signaling molecules acetylcholine and (nor-) epinephrine. Pharmacological modifiers of these pathways are widely used in the clinical setting, especially during and after surgery and sepsis. As anesthesiologists and perioperative physicians, we are especially interested in investigating how the use of these agents influences the underlying diseases leading to surgery. Clinical studies implicate that the long-term outcome of malignant diseases is substantially influenced by the modulation of tumor immunosurveillance during “stress” responses and medical treatments. While it is widely appreciated that “immuno-stimulatory” compounds such as CpG-ODN can elicit potent antitumor immune responses (for a detailed review see [33]), the effects of systemic inflammation and the subsequent treatment on tumor immuno-surveillance are understudied. In a study by Laurent and colleagues on the long-term outcome of total mesorectal excision for rectal cancer, overall 5-year survival and disease-free 5-year survival were significantly decreased in septic patients (odds ratio 2.06 and 2.17) [34]. Local and distant tumor recurrence were significantly increased in these patients (local recurrence: 11% vs. 5% (septic vs. non-septic), distant recurrence: 32% vs. 23% (septic vs. non-septic)). Tan and colleagues reported similar findings: In their study on surgically treated renal cell carcinoma, sepsis was an independent risk factor negatively influencing long-term survival [35]. Other clinical studies in patients with different kind of cancers support these results demonstrating poorer survival or increased tumor recurrence in septic patients or patients with postoperative infections [36], [37], [38]. These findings have to be interpreted carefully. Sepsis is thought to be accompanied by an initial “hyperimmune” and a subsequent “hypoimmune” state [39], both of which could have an impact on tumor immunosurveillance. Sepsis treatment includes the use of antibiotics, vasopressors, volume resuscitation and the transfusion of blood products, all of which are known to modulate various aspects of immune responses. Last not least, we would like to point out that ADRB2-mediated suppression of inflammation could be a desirable side effect of treatment. This is reflected by the standard use of epinephrine in anaphylaxis, as stated in the current ILCOR/AHA/ERC guidelines on cardiopulmonary resuscitation (2010).

In summary, we provide insights into the underlying mechanisms of the interrelation between immune responses and pharmacological agents which are widely used in the medical treatment of human patients, also during or after oncologic surgery. Future studies have to investigate the *in vivo* relevance of these findings. Our results have implications for the future treatment of human patients, in which the endogenous immune response plays a pivotal role, such as during viral infections, inflammatory diseases and cancers.
